# Creation of discontinuities in circle maps

**DOI:** 10.1098/rspa.2020.0872

**Published:** 2021-07

**Authors:** G. Derks, P. A. Glendinning, A. C. Skeldon

**Affiliations:** ^1^ Department of Mathematics, University of Surrey, Guildford GU2 7XH, UK; ^2^ Department of Mathematics, University of Manchester, Oxford Road, Manchester M13 9PL, UK

**Keywords:** bifurcation, circle map, threshold model, Cherry flow, discontinuous map, sleep–wake regulation, neuronal models

## Abstract

Circle maps frequently arise in mathematical models of physical or biological systems. Motivated by Cherry flows and ‘threshold’ systems such as integrate and fire neuronal models, models of cardiac arrhythmias, and models of sleep/wake regulation, we consider how structural transitions in circle maps occur. In particular, we describe how maps evolve near the creation of a discontinuity. We show that the natural way to create discontinuities in the maps associated with both threshold systems and Cherry flows results in a singularity in the derivative of the map as the discontinuity is approached from either one or both sides. For the threshold systems, the associated maps have square root singularities and we analyse the generic properties of such maps with gaps, showing how border collisions and saddle-node bifurcations are interspersed. This highlights how the Arnold tongue picture for tongues bordered by saddle-node bifurcations is amended once gaps are present. We also show that a loss of injectivity naturally results in the creation of multiple gaps giving rise to a novel codimension two bifurcation.

## 1. Introduction

**Figure 1.**
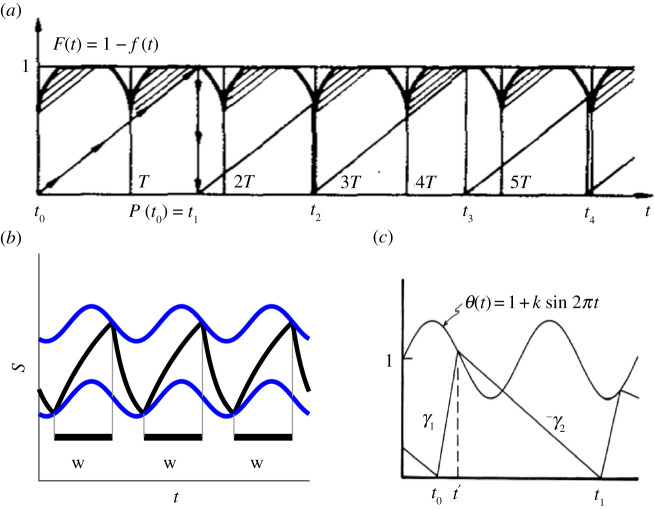
(*a*) A model of cardiac arrhythmias, attributed to Gel’fand and Tsetlin. Reprinted from [1] with the permission of AIP Publishing. (*b*) The two-process model of sleep–wake regulation; sketch based on the model in [6]. (*c*) An integrate and fire model. Reprinted from [2] with the permission of AIP Publishing. This model will be described in detail in §2a and be called the sinusoidal threshold system (STS). (Online version in colour.)

Circle maps arise in many mathematical models of real-life systems. Our motivation for this paper comes from two classes of models. The first arises in many biological models including models of cardiac arrhythmias (see [1,2] and references therein), neuronal models [3,4] and the two-process model of sleep–wake regulation [5,6]. We refer to these examples as ‘threshold systems’, since in each case, a variable of interest increases until it hits an upper threshold, decreases until it hits a lower threshold and then repeats. Some typical examples are shown in figure 1. If the thresholds are periodic with the same period, then each system can be represented by a circle map [2,7,8] and the resulting observed phenomena include phase-locked solutions, ‘period-adding’, period-doubling and chaos.

The second class of models has found application in problems of breathing rhythms [9] and arises naturally in coupled oscillator problems at appropriate parameter values [10]. The initial model is a flow on the torus. If there are no stationary points and a global cross-section (a Poincaré flow), then the return map on this section is a continuous monotonic circle map. If, as parameters are varied, a pair of stationary points is created by saddle-node bifurcations then the resulting flow is known as a Cherry flow [11]. These can generate return maps which have either discontinuities or regions where the map is not defined [12].

Degree one circle maps $f:S^{1}\to S^{1}$ are described by real functions F:R→R with *F*(*x* + 1) = *F*(*x*) + 1 and *f*(*x*) = *F*(*x*) modulo 1. These maps arise in many situations and *F* may be continuous (or not) and injective (or not) leading to four different types of map: *f* is continuous and injective (monotonic), the classic case; continuous and non-injective [13]; discontinuous and injective [2]; and discontinuous and non-injective [8]. In many applications, the type of map is fixed. However, for maps derived from the classes of models considered above changes of type occur naturally as parameters vary. Close to the transitions between types, the maps have a well-defined structure. Parameter changes in this structure in turn may change some dynamical properties of the systems. Note that the transition between types does not necessarily imply the existence of a local bifurcation of attractors. However, these transitions do have consequences for the possible dynamics, and these consequences are the subject of this paper.

There is a vast literature on circle maps that are both continuous and injective (monotonic) (e.g. [14]). All points have the same rotation number (average rate of rotation) under iteration by such maps. If the rotation number is rational then solutions tend to periodic orbits. Whilst if the rotation number is irrational there are no periodic solutions and if the map is sufficiently smooth (e.g. *C*^2^), all orbits are dense in the circle. Deeper results about the smoothness of conjugacies to rigid rotations for maps with irrational rotation numbers were developed by Herman [15] and led to many technical results in this direction [16]. For typical families of circle maps, the rotation number takes rational values on closed intervals of parameters. This is called mode-locking and the regions of parameter space with a given rational rotation number is an Arnold tongue. The Arnold tongues are bounded by saddle-node bifurcations.

If the circle map is continuous but not monotonic then the rotation number is replaced by a rotation interval [17]. Many properties can be understood using classic results for maps of the interval and the transition from continuous and monotonic circle maps to continuous and non-monotonic maps has been described in some detail, including the transitions to the chaos which involve different sequences of period-doubling bifurcations [18–20].

The injective and discontinuous circle maps arise in a number of contexts and basic results such as the existence of a well-defined rotation number can be found in [21–23]. The review paper [24] gives a thorough summary of the current literature on these monotonic circle maps with gaps, i.e. intervals with no pre-images, and shows how maps of the real line with gaps can be framed as circle maps. This is particularly important because it highlights how many results known from the study of circle maps have been rediscovered in the context of maps of the real line.

The non-injective discontinuous circle maps can be divided into sub-classes. If the continuous branches are increasing, then this includes the Lorenz maps and again a lot is understood (e.g. [25]). Less is known about the details of the dynamics of non-injective discontinuous maps in general (although the techniques of kneading theory do apply), partly because it is less clear what results would be useful without further context.

Both threshold systems and the transition from Poincaré flows to Cherry flows provide natural settings to consider the transition from continuous circle maps to piecewise continuous circle maps with discontinuities. In each case, specific properties of the original dynamical system induce transitions between different circle map types. For the transition to discontinuous maps in both of these cases, we show that the derivative of the map is singular on at least one side of the discontinuity and derive scaling results. Although the derivative becomes singular, derivatives may be large for a small neighbourhood of the singular value only and so can be difficult to resolve numerically. Nevertheless, we show that the presence of the singularity is essential to understanding how a continuous circle map with phase-locked regions bounded by saddle-node bifurcations transitions to a circle map with a gap, with creation/destruction of periodic solutions via border collisions and saddle-node bifurcations. We further show that in threshold systems there is a natural route to creating maps with multiple gaps that result in a novel codimension two bifurcation similar to the ‘big bang’ bifurcation seen in other contexts [26].

The layout of the paper is as follows. In §2, we define smooth threshold systems and the associated circle maps. An extension of the standard Arnold map is presented as an example. In §3, we discuss the creation of gaps in smooth threshold systems, deriving the typical scalings for the gap size and showing that the map to one side of the gap has a square-root singularity. In §4, we consider a general form for a piecewise continuous map with a gap on which one side the map has a square-root singularity and discuss two examples. In §5, we discuss the creation of non-monotonicity in threshold systems and how this can result in multiple gaps and lead to codimension two bifurcations. In §6, we consider Cherry flows and discuss the creation of a discontinuity (cf. [27]), showing that a finite gap is created instantaneously and the slope of the return map has a square-root singularity. We end with a short discussion.

## 2. Threshold systems

The essential feature of a threshold system is that there is a dependent variable which increases until it hits an upper threshold, decreases until it hits a lower threshold and then repeats. The following definition formalizes this idea in the case of smooth thresholds which provide the generic cases described later in this paper. In this definition, *x* represents the independent time-like variable, and for any flow $\phi_{x}:R\to R$, *ϕ*_*x*_ depends smoothly on the independent variable *x*, *ϕ*_0_ is the identity and for all r,s∈R, $\phi_{r}\circ \phi_{s}=\phi_{r+s}$.

**Definition 2.1.**

A smooth threshold system is a pair of flows *ϕ*_*x*_ and *ψ*_*x*_, the upflow and downflow, respectively, and an upper threshold and a lower threshold such that

(i) The upflow is strictly increasing and the downflow is strictly decreasing.
(ii) The upper and lower thresholds are the graphs of smooth real functions *y* = *h*(*x*) and *y* = *g*(*x*) with period one, respectively, such that for all x∈R
  2.1 g(x)<h(x).
(iii) Starting from the lower threshold, the upflow reaches the upper threshold in finite time and vice versa. Formally, if *y*_0_ = *g*(*x*_0_), then there exists *τ* > 0 such that *ϕ*_*τ*_(*y*_0_) = *h*(*x*_0_ + *τ*), and if *y*_0_ = *h*(*x*_0_) then there exists *τ*^′^ > 0 such that $\psi_{\tau^{\prime }}(y_{0})=g(x_{0}+\tau^{\prime })$.

To determine the dynamics of a threshold system consider an initial condition on the upper threshold, (*x*_*n*_, *y*_*n*_) with *y*_*n*_ = *h*(*x*_*n*_). By property (iii), there is a smallest *τ*_*n*_ > 0 such that $\left(x_{n}+\tau_{n},{\tilde{y}}_{n}\right)$ is on the lower threshold with ${\tilde{y}}_{n}=\psi_{\tau_{n}}(y_{n})=g(x_{n}+\tau_{n})$. Now using the upflow part of property (iii), there exists a smallest $\tau_{n}^{\prime }>0$ such that

$$y_{n+1}:=\phi_{\tau_{n}^{\prime }}({\tilde{y}}_{n})=h(x_{n}+\tau_{n}+\tau_{n}^{\prime }).$$

Thus, starting at *x*_*n*_ on the upper boundary, the trajectory returns to the upper boundary at time $x_{n}+\tau_{n}+\tau_{n}^{\prime }$, generating a map

$$x_{n+1}=F(x_{n})=x_{n}+\tau_{n}+\tau_{n}^{\prime }.$$

If the trajectory had started at the upper boundary with *x*-coordinate *x*_*n*_ + 1 then the periodicity of *g* and *h* implies that the return to the upper boundary would be at $x_{n}+1+\tau_{n}+\tau_{n}^{\prime }$ and so *F*(*x* + 1) = *F*(*x*) + 1, thus *F* is the lift of a degree one circle map.

In §§3 and 5, we will show that the monotonicity and continuity of the circle map of a smooth threshold system are related to the absence of tangencies between the thresholds and flows. Throughout this paper, we illustrate our findings with the standard example of a sinusoidal threshold, sketched in figure 1*c* and described below. The section below also contains the Arnold tongue structure for this example.

### (a) Example: the sinusoidal threshold system

The example shown in figure 1*c* we refer to as the sinusoidal threshold system (STS). We believe that this dynamical system first appeared explicitly in a paper by Glass and Belair in 1986 [28]. In a 1991 paper [2], Glass refers to it as the Gel’fand and Tsetlin integrate and fire model, although he was unable to locate a reference. He notes that it is also studied by Arnold in his 1959 thesis, an excerpt of which is in [1], although no explicit model is given. Although the STS appears in [2], the dynamics were not fully analysed. In [28], three special cases are considered: infinite slope for the downflow (reset dynamics), equal rates for the upflow and downflow and infinite slope for the upflow. The first and last cases seem to be investigated in detail in various papers, but the middle one is referred to as ‘hope to investigate later’. The case with the infinite slope in the downflow is considered by Winfree in [29] in the context of the entrainment of circadian rhythms. The STS can also be thought of as a simplified form of the two-process model of sleep–wake regulation [5,6].

For the STS, the upper and lower thresholds are given by the functions

2.2 $$\begin{array}{rl} & y=h(x)=\beta +\frac{\alpha }{2\pi }(1+\sin2\pi x)\\ \text{and}\; & y=g(x)=0, \end{array}\}$$

respectively, with *α* > 0 and *β* > 0. The upflow and downflow are linear functions as shown in figure 1*c*: *ϕ*_*x*_(*y*_0_) = *y*_0_ + *γ* *x*, *γ* > 0 (i.e. the solution to d*y*/d*x* = *γ*) and *ψ*_*x*_(*y*_0_) = *y*_0_ − *x* (the solution to d*y*/d*x* = −1). This system is equivalent to the system in [2] with a rescaling of the parameters.

The derivation of the associated circle map then follows. Suppose that at *x*_*n*_, the system is on the upper threshold, with *y*_*n*_ = *h*(*x*_*n*_). Then, the trajectory of the downflow will reach the lower threshold after additional time *τ*_*n*_, where *τ*_*n*_ = *h*(*x*_*n*_), and the new value of the independent variable is ${\tilde{x}}_{n}=x_{n}+\tau_{n}$ with *y*-coordinate equal to zero. The time taken to return to the upper threshold using the upflow is $\tau_{n}^{\prime }$, which is determined implicitly by

2.3 $$\gamma \tau_{n}^{\prime }=h(x_{n}+\tau_{n}+\tau_{n}^{\prime }).$$

If (2.3) has multiple positive solutions then the smallest possible *τ*^′^ is chosen. The return map to the upper threshold is therefore $x_{n+1}=F(x_{n})=x_{n}+\tau_{n}+\tau_{n}^{\prime }$ or (as an implicit difference equation)

2.4 $$x_{n+1}=x_{n}+h(x_{n})+\frac{1}{\gamma }h(x_{n+1}).$$

An immediate consequence is that as *γ* → ∞ (a classic reset) this reduces to the classic Arnold (sine) circle map [1,2] which has been extensively studied.

The gradient of the map determines properties such as monotonicity. Implicit differentiation of (2.4) gives

2.5 $$\frac{\text{d}x_{n+1}}{\text{d}x_{n}}=\frac{1+h^{\prime }(x_{n})}{1-(1/\gamma )h^{\prime }(x_{n+1})}.$$

Since *h*^′^ (*x*) = *α*cos (2*πx*), the numerator of (2.5) is always positive if *α* < 1 (recall *α* is positive) and the denominator is positive provided *α* < *γ*. In particular, the map is monotonic and continuous if *α* < min (1, *γ*).

The derivative of (2.5) becomes singular when the upflow becomes tangent to the upper threshold. In §3, we will show that this is a generic feature in maps generated by threshold systems and describe the generic development of a discontinuity and singular derivative in the map. Similarly, (2.5) shows that the derivative vanishes when the downflow becomes tangent to the upper boundary. In §5, we will show that such a tangency generates non-monotonicity in the map generated by a threshold system. Thus, the STS example illustrates the generic transition from monotonicity to non-monotonicity when *α* = 1 and the generic transition from continuous to non-continuous when *α* = *γ*.

One attractive feature of the STS is that explicit expressions for some of the bifurcations can be found. A periodic solution on the circle corresponds to a solution of the form *F*^*q*^(*x*) = *x* + *p* and has rotation number *p*/*q*. We will refer to such solutions as (*p*, *q*)-periodic orbits, and they satisfy

2.6 $$x_{n+q}=x_{n}+p.$$

In the STS, a necessary condition for the existence of (*p*, 1)-periodic solutions is that there exists *x* such that

2.7 $$\sin2\pi x=\frac{2\pi }{\alpha }(p\tilde{\gamma }-\beta )-1,\;\text{with}\;\tilde{\gamma }=\frac{\gamma }{1+\gamma }.$$

As we will see in §3, this condition is not sufficient if the map has a discontinuity. This is related to the fact that a threshold system is defined on the first intersection of the up/downflow with the upper/lower threshold. Solutions to (2.7) exist provided its right-hand side has modulus less than or equal to one. Thus for fixed *γ*, the maximal region of existence of (*p*, 1)-periodic solutions in the (*β*, *α*) parameter plane with *α* > 0 is bounded by the curves

$$\alpha =\pi (p\tilde{\gamma }-\beta ),\;\text{and}\;\beta =p\tilde{\gamma },$$

on which *x* takes the values 1/4 and 3/4, respectively. If these *x* values correspond to first intersections of the upflow with the upper threshold (as is the case when the map is continuous, i.e. for *α* < *γ*), these curves are lines of saddle-node bifurcations that create one stable and one unstable periodic solution. They form tongue-like regions emanating from *α* = 0, $\beta =p\tilde{\gamma }$.

**Figure 2.**
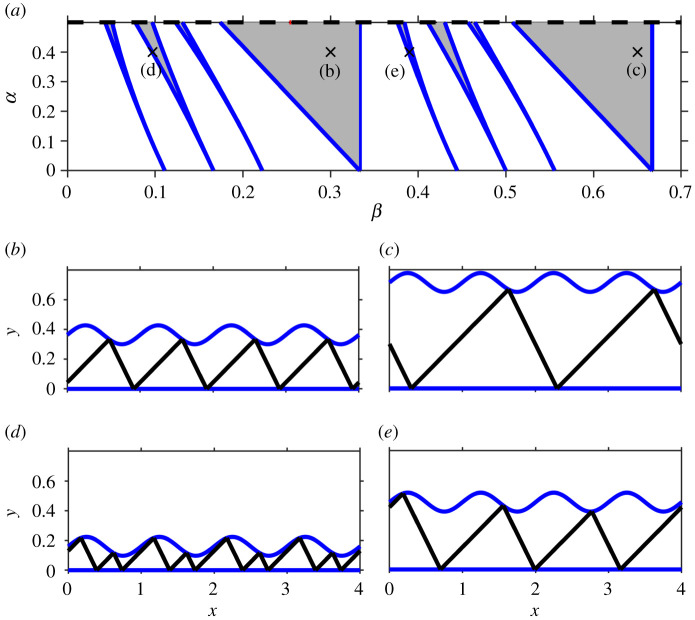
(*a*) Bifurcation set showing the largest few tongues that bound the regions of existence for periodic solutions (*γ* = 0.5). The blue lines are lines of saddle-node bifurcations. (*b*–*e*) Trajectories for periodic solutions with (*p*, *q*) = (1, 1), (2, 1), (1, 2) and (4, 3), respectively (*α* = 0.4, *γ* = 0.5 and *β* = 0.3, 0.65, 0.097 and 0.39, respectively). (Online version in colour.)

Saddle-node bifurcations for general (*p*, *q*) tongues can be found by numerically solving equation (2.6) along with the condition that the gradient of the *q*th iterate of the map is one. Typical Arnold tongues for the STS with *α* < *γ* are shown in figure 2. We note that there is a parameter symmetry for existence regions for periodic solutions. Specifically, if for some *α*, *β*, *γ*, there exists a (*p*, *q*)-periodic orbit, where $p,q\in Z^{+}$, and *p* and *q* are relatively prime, then there also exists a $\left(\tilde{p},q\right)$-periodic orbit, where $\tilde{p}=p+mq$, $m\in Z^{+}$ for *α*, *γ* and

$$\tilde{\beta }=\beta +\frac{m\gamma }{1+\gamma }=\beta +m\tilde{\gamma }.$$

This symmetry is then reflected in the positions of the tongues: in figure 2, the (2, 1)-tongue is a translation of the (1, 1)-tongue, the (3, 2)-tongue is a translation of the (1, 2)-tongue, the (4, 3)-tongue is a translation of the (1, 3)-tongue and the (5, 3)-tongue is a translation of the (2, 3)-tongue. We note that in [2] the different choice of scaling for the STS means that the parameter symmetry is not immediately evident in the computed bifurcation sets.

At *α* = *γ*, the upflow becomes tangent to the upper threshold and the map loses smoothness at the pre-image of this tangency point (see (2.5)). As we will show in the next section, the map develops a discontinuity for *α* > *γ*. We will continue with this example after deriving the general theory.

## 3. Tangencies leading to gaps

**Figure 3.**
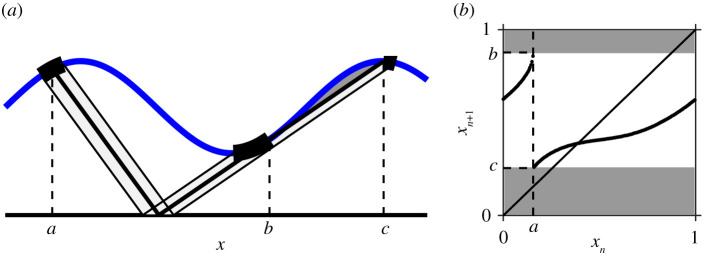
(*a*) A tangency of the upflow with the upper threshold for the STS (*α* = 0.7, *β* = 0.15, *γ* = 0.5). The tangency occurs at the point *x* = *b* which has a pre-image at *x* = *a*. The figure illustrates how the region local to *a* maps to two disjoint sets, one local to *b* and one local to *c*, where *x* = *c* is the position of the second intersection. The shadow region is shaded in dark grey and corresponds to *x* ∈ (*b*, *c*]. (*b*) Corresponding circle map. (Online version in colour.)

As shown by Arnold [1], gaps in a threshold map are a result of ‘shadow’ regions in the dynamics, that is, regions for which the upper threshold is unreachable, as illustrated in figure 3*a* for the STS. In this example, there are sections of the upper threshold such that no trajectory from the lower threshold can reach this section without first having crossed the upper threshold at least once before. These ‘shadow’ regions are then reflected as gaps in the associated circle map, as shown in figure 3*b*. For sufficiently smooth flows and thresholds, the generic transition from no gaps to gaps will occur as a result of tangency of either the upflow with the upper threshold or the downflow with the lower threshold.

In this section, we will look at parametrized families of threshold systems. We determine generic criteria for the creation of a discontinuity and describe the local behaviour nearby. The construction of the return map involves solving for the zeros of a function which can be treated in almost precisely the same way as the standard bifurcation theory for fixed points (e.g. [30, chs 4 and 8]). Let P⊂R be the parameter space. Parametrized threshold maps can be thought of as the composition of two maps: the down map $T_{d}:R\times P\to R$ from the upper boundary to the lower boundary and the up map $T_{u}:R\times P\to R$ from the lower boundary to the upper boundary as described in §2 but with the addition of a real parameter, so *T*_*u*,*d*_ = *T*_*u*,*d*_(*x*, *μ*). The periodicity of the thresholds implies that both of these maps have period one in the first variable: *T*_*u*,*d*_(*x* + 1, *μ*) = *T*_*u*,*d*_(*x*, *μ*) for all x∈R.

Assume that the down map is a smooth bijection of the real line for all *μ* in the region of interest. Thus, the down trajectory from (*x*_*n*_, *h*(*x*_*n*_, *μ*)) will intersect the lower threshold at (*x*, *g*(*x*, *μ*)) with *x* = *T*_*d*_(*x*_*n*_, *μ*) and for every x∈R such an *x*_*n*_ exists.

The trajectory under the upflow *ϕ* starting at (*x*, *g*(*x*, *μ*)) on the lower threshold will intersect the upper threshold *y* = *h*(*x*, *μ*) at time(s) *τ* which satisfy

3.1 $$W(\tau ,x,\mu ):=\phi_{\tau }(g(x,\mu ),\mu )-h(x+\tau ,\mu )=0.$$

We will be interested in local behaviour near a solution (τ*, x*, μ*) of (3.1) representing a first intersection of the upflow with the upper boundary. By shifting coordinates we may choose (x*, μ*) = (0, 0), and from now on, we assume this shift of coordinates has been implemented. The essential genericity condition on the *x* and *μ* behaviour, assumed throughout this section, is that

3.2 $$W_{2}(\tau^{*},0,0)\neq 0\;\text{and}\;W_{3}(\tau^{*},0,0)\neq 0.$$

Here, we used subscripts to denote partial differentiation by the *i*th variable, e.g. *W*_2_ = ∂*W*/∂*x*.

**Figure 4.**
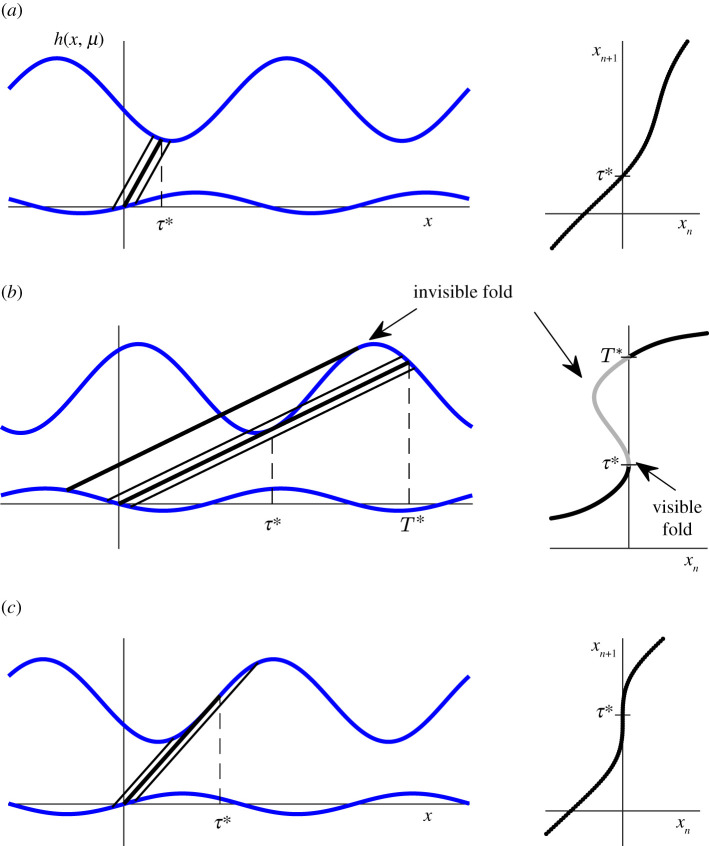
Tangencies leading to gaps. (*a*) Unique solution to τ*. (*b*) Existence of a simple tangency between the upflow and the upper boundary. (*c*) The cusp catastrophe. (Online version in colour.)

The map is locally continuous if in addition *W*_1_(τ*, 0, 0) ≠ 0. Indeed, a standard application of the implicit function theorem yields a unique and smooth local solution *τ*(*x*, *μ*) to (3.1) of the form

$$\tau =\tau^{*}-x\;\frac{W_{2}}{W_{1}}-\mu \;\frac{W_{3}}{W_{1}}+O(2),$$

where the partial derivatives are evaluated at (τ*, 0, 0) (see figure 4*a*).

There is a simple tangency between the upwards trajectory and the upper boundary if

3.3 $$W(\tau^{*},0,0)=0,\;W_{1}(\tau^{*},0,0)=0,\;W_{11}(\tau^{*},0,0)\neq 0$$

(see figure 4*b*). Although this is not the onset of discontinuities, it is worth considering as it shows the persistence of jumps in the one-dimensional map. In this case, the intersection equation (3.1) can be written locally as

3.4 $${\left(\tau -\tau^{*}\right)}^{2}=-\frac{2}{W_{11}}(W_{2}x+W_{3}\mu )+O(2).$$

Hence, there is a fold along a curve in the (*x*, *μ*)-plane given by *W*_2_*x* + *W*_3_*μ* = 0 in the lowest order. The fold has two interpretations: it represents a unique local solution to (3.1) (a fold point) *τ*(*μ*) = τ* + *O*(*μ*). In the threshold system, it gives also a persisting simple tangency between the upwards trajectory and the upper threshold at this point. When *W*_11_(*W*_2_*x* + *W*_3_*μ*) < 0, then (3.1) has locally two solutions, one less and one greater than τ*. The map is defined using the negative solution to (3.4) as this represents the first intersection of the upflow and the upper threshold. Locally, the second solution does not play a role in defining the map. Furthermore, the derivative ∂*τ*/∂*x* = (*W*_2_/*W*_11_)(τ* − *τ*) in lowest order, hence for (*x*, *μ*), with *W*_11_(*W*_2_*x* + *W*_3_*μ*) < 0, approaching the fold curve, the derivative of the map becomes unbounded at τ* and exhibits a square-root singularity.

By definition 2.1, there are solutions to *W*(*τ*, *x*, *μ*) = 0 even if *W*_11_(*W*_2_*x* + *W*_3_*μ*) > 0. Generically, this implies that (3.1) has a second (typically non-local) solution (T*, 0, 0), T* > τ*, representing the second intersection between the upflow and the upper threshold (after the first one at (τ*, 0, 0)). Generically, all the first derivatives at (T*, 0, 0) are non-zero and the map can be continued for *W*_11_(*W*_2_*x* + *W*_3_*μ*) > 0, with a discontinuity and finite derivative on approaching the fold curve *W*_2_*x* + *W*_3_*μ* = *O*(2) from this side.

This proves that once the jump exists, it is stable to perturbations of the map. This analysis also implies that there is a second fold between τ* and T* for some fold curve (*x*, *μ*) with *W*_11_(*W*_2_*x* + *W*_3_*μ*) < 0. This fold is not part of the return map, and at the risk of confusing with the standard names in piecewise-smooth dynamics (of which this is a natural counterpart), we refer to this second fold as an invisible fold and to the fold at τ* as a *visible* fold (see figure 4*b*).

So at a standard fold satisfying (3.3), the discontinuity already exists (T* of the previous paragraphs is non-local). To obtain the transition from continuity to discontinuity, we need a further condition:

3.5 $$W(\tau^{*},0,0)=W_{1}(\tau^{*},0,0)=W_{11}(\tau^{*},0,0)=0,\;W_{111}(\tau^{*},0,0)\neq 0$$

(see figure 4*c*). Taken together with the genericity condition (3.2) this defines a standard unfolding of the cubic singularity: a cusp catastrophe. The standard form for the unfolding of the cusp is

3.6 $$A+Bu+u^{3}=0,\;u=\tau -\tau^{*},$$

which has three solutions if *A*^2^ < (4/27)|*B*|^3^, *B* < 0, and otherwise, one solution except on the curves *A*^2^ = (4/27)|*B*|^3^, *B* < 0, which are the loci of local folds. Using the Taylor series for *W*, the intersection equation (3.1) becomes

$$\begin{array}{rl} 0 & =(W_{2}x+W_{3}\mu +O{\left((|x|+|\mu |\right)}^{2}))+(W_{12}x+W_{13}\mu +O{\left((|x|+|\mu |\right)}^{2}))u\\ & \;+\frac{1}{2}(W_{112}x+W_{113}\mu +O{\left((|x|+|\mu |\right)}^{2}))u^{2}+\frac{1}{6}(W_{111}+O(|x|+|\mu |))u^{3}+O(u^{4}). \end{array}$$

The transformation *u* → *u* − (1/*W*_111_)(*W*_112_*x* + *W*_113_*μ*) transforms this to

3.7 $$0=a(x,\mu )+b(x,\mu )u+\left(1+O(|x|+|\mu |)\right)u^{3}+O(u^{4}),$$

where

3.8 $$a(x,\mu )=\frac{6}{W_{111}}(W_{2}x+W_{3}\mu )+O({\left(|x|+|\mu |\right)}^{2})$$

and

3.9 $$b(x,\mu )=\frac{6}{W_{111}}(W_{12}x+W_{13}\mu )+O{\left((|x|+|\mu |\right)}^{2}).$$

Note that *a*(0, 0) = *b*(0, 0) = 0. Provided the transformation from (*a*, *b*) to (*x*, *μ*) is non-singular, i.e. provided

3.10 $$\det\left(\frac{\partial (a,b)}{\partial (x,\mu )}\right){|}_{\left(0,0\right)}\neq 0,$$

all the standard results for the unfolding of the *x*^3^ singularity in the (*a*, *b*) plane carry over to the (*x*, *μ*) plane. A glance at the standard cusp manifold shows that one of the branches of the folds defined by the cusp is invisible, and the other is visible, creating a bifurcation curve of jumps in the (*x*, *μ*) plane terminating at (0, 0).

**Lemma 3.1.**

*Let a*(*x*, *μ*), *b*(*x*, *μ*) *be as defined in* (3.8) *and* (3.9). *Suppose that the nondegeneracy condition* (3.2) *and the double tangency condition* (3.5) *hold with*

3.11 $$W_{2}W_{13}-W_{3}W_{12}\neq 0.$$

*Then, for σ* ∈ { + 1, − 1}, *the locus* $a=\sigma (2/3\sqrt{3})|b{|}^{3/2}$, *b* < 0, *gives a fold at* $u=\sigma \sqrt{\frac{|b|}{3}}$.

**Proof.**

Equation (3.11) is just a rewriting of (3.10). The remainder is a restatement of properties of the standard unfolding of the singularity ±*u*^3^. ▪

Lemma 3.1 is a re-interpretation of the cusp bifurcation (see [30, §8.2]). The up map *T*_*u*_ is

$$x\to x+u_{m}+\tau^{*}-\frac{W_{112}x+W_{113}\mu }{W_{111}},$$

where *u* = *u*_*m*_ is the smallest *u* value satisfying (3.7); hence, the visible fold corresponds to *σ* = −1 in lemma 3.1. The lemma determines the locus of the discontinuity but not the effect.

**Proposition 3.2.**

*Suppose that the down map T*_*d*_ *is a smooth bijection and that* (3.2), (3.5) *and* (3.11) *hold. Then, the corresponding up map T*_*u*_ *and the threshold map* $T=T_{u}\circ T_{d}$ *have a discontinuity of size of order* $\sqrt{|\mu |}$ *on the visible fold line*.

**Proof.**

The size of the discontinuity is the change in *τ* values from the value on the visible fold to the other value of the cubic (3.7). On the visible fold *u*^2^ = −(1/3)*b* > 0 to lowest order, and if we choose the negative solution, $u=-\sqrt{(-b)/3}$ then substituting back into (3.7) gives $a=-(2/3\sqrt{3})|b{|}^{3/2}$ [30]. At the fold, there are only two distinct solutions. The solution $u\approx -\sqrt{(-b)/3}$ already discussed is a repeated root, and so the other solution can be found by solving ${\left(u+\sqrt{(-b)/3}\right)}^{2}(u-\alpha )=a+bu+u^{3}$ to give $\alpha =2\sqrt{(-b)/3}$ to lowest order. The jump is therefore the difference of the two roots, i.e. $3\sqrt{(-b)/3}$.

On the fold *a*^2^ = −(4/27)*b*^3^, with *a* and *b* linear functions of the original variables *x* and *μ* via (3.8) and (3.9). To lowest order, this implies that the fold is tangential to the line *a* = 0, i.e. *x* ∼ −*W*_3_*μ*/*W*_2_ and so the evaluating *b* on this line the jump becomes

$$3\sqrt{2|\frac{W_{12}W_{3}-W_{13}W_{2}}{W_{2}W_{111}}\mu |}.$$

Thus, the jump is order $\sqrt{|\mu |}$.

Note that although we have derived these properties for the up map alone, they are preserved by composition with a smoothly bijective down map. ▪

## 4. Square-root discontinuities in monotonic circle maps

The discontinuous circle maps derived in §3 have two features: there is an interval of values which cannot be reached by the map, and on one side of this gap, the derivative of the map tends to infinity with a square-root singularity. In this section, we will consider the fundamental bifurcation sequences for periodic solutions of the simplest form of such maps and illustrate the observations with two examples.

In terms of the lift *F* of such a circle map, defined on the real line with *F*(*x* + 1) = *F*(*x*) + 1, coordinates can be chosen such that

$$\lim_{x↓0}F(x)+1>\lim_{x↑1}F(x),$$

and *F* is continuously differentiable and strictly increasing on (0, 1). In other words, the gap is a jump upwards at integer values of *x*. The square-root discontinuity implies that

$$\lim_{x↓0}F^{\prime }(x)=c>0,\;F^{\prime }(x)\to \infty \;\text{as}\;x↑1,$$

or vice versa. Thus, *F* is strictly increasing and hence has a unique rotation number [22].

Now consider families $\left(F_{\mu }\right)$ of such maps which are continuous in *μ* and such that $F_{\mu }$ is an increasing function of *μ* for each *x*. The continuity in *μ* implies that the rotation number is a continuous function of *μ* (see [23, theorem 5.8]). Since $F_{\mu }$ is an increasing function of *μ*, the rotation number is monotonic in *μ*, irrational values of the rotation number exist at isolated values of *μ* (see [23, proposition 6.1]) and the invariant set for an irrational rotation number is a Cantor set [21]. Finally, under the same conditions, the set of *μ* with a given rational rotation number is a non-trivial closed interval, i.e. the maps have phase-locking analogous to that of continuous homeomorphisms of the circle (see [23, theorem 6.6]).

The gaps introduce a second mechanism by which phase-locked solutions can be created or destroyed, that is via border collisions. We will call a border collision with an endpoint with an infinite derivative a *type I* border collision and use *type II* for a border collision with an endpoint with a finite derivative. Within each phase-locked region, there is a branch of periodic solutions connecting 0^+^ with 1^−^. If $F_{\mu }^{\prime }(x)<1$ for all *x* ∈ (0, 1), then there are two border collision bifurcations, one creating a stable fixed point and the other destroying it and similarly for $F_{\mu }^{q}$ in a *p*/*q* phase-locked region. There can be no saddle-node bifurcations in such maps. However, for maps with a square-root singularity, $F_{\mu }^{\prime }(x)$ cannot be less than one for all *x* ∈ (0, 1), although to remain an injection $F_{\mu }^{\prime }(x)<1$ for some *x* ∈ (0, 1). For maps $F_{\mu }(x)$ that are monotonic increasing in both *x* and *μ*, the type I border collision with the square-root singularity at 1^−^ always leads to the creation of an unstable periodic orbit when *μ* is increased. The type II border collision with 0^+^ can lead to either the creation of a stable periodic orbit (if ${\left(F_{\mu }^{q}\right)}^{\prime }(0^{+})<1$) or the loss of an unstable periodic orbit (if ${\left(F_{\mu }^{q}\right)}^{\prime }(0^{+})>1$).

There appear to be two possible ‘simplest’ sequences of bifurcations for the creation/ destruction of periodic orbits of a given rotation number *p*/*q*:

(a) border collision → border collision → saddle-node bifurcation;
(b) saddle-node bifurcation → border collision → border collision → saddle-node bifurcation.

**Figure 5.**
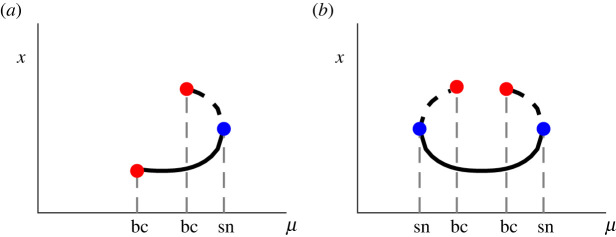
Bifurcation diagrams illustrating the two sequences: (*a*) border collision → border collision → saddle-node bifurcation; (*b*) saddle-node bifurcation → border collision → border collision → saddle-node bifurcation. (Online version in colour.)

These two bifurcation sequences are illustrated in the bifurcation diagram shown in figure 5. Case (a) occurs if ${\left(F_{\mu }^{q}\right)}^{\prime }(0^{+})<1$ when first a stable periodic orbit is created in a border collision at 0^+^, then a second unstable periodic orbit is created in a border collision at 1^−^ and finally, both solutions disappear in a saddle-node bifurcation. Case (b) occurs if ${\left(F_{\mu }^{q}\right)}^{\prime }(0^{+})>1$. In both bifurcation sequences, there is always a stable periodic solution in each phase-locked region. Of course, the bifurcation sequences could have extra pairs of saddle-node bifurcations. However, if $F_{\mu }$ is convex, then also $F_{\mu \mu }^{q}$ is piecewise convex and case (a) will always occur as the convexity implies that ${\left(F_{\mu }^{q}\right)}^{\prime }$ is monotonic increasing, hence at most one saddle-node bifurcation is possible. Note also that $F_{\mu }^{\prime }(0^{+})≶1$ does not imply that ${\left(F_{\mu }^{q}\right)}^{\prime }(0^{+})≶1$ for all *q* > 1, so it may be possible to find both case (a) and case (b) in examples.

The monotonicity of the map in the parameter ensures that structures are not repeated; if this parameter monotonicity assumption is not true, the essential results hold true but there may be multiple parameter values with a given irrational rotation number (or even intervals) and the bifurcation structure of phase-locked regions can be considerably more complicated.

In the next sections, we illustrate the bifurcation sequences (a) and (b) with a ‘canonical’ example and then show how they are manifested in the STS.

### (a) Example: ‘canonical’ square-root singularity maps

To illustrate the consequences of the square-root singularity, we have constructed an example which is monotonic in *x* and its parameters and demonstrates the bifurcation sequences described earlier. This example can be derived from threshold models, by using the inverse to define an upper threshold with the gap replaced by a smooth continuation and taking the lower threshold to be a constant with *T*_*d*_ equal to the identity. For *n* ≥ 2, define the lift *F*_*n*_ by

4.1 $$F_{n}(x)=a+b\left(1-c_{1}\sqrt{1-x}+(c_{1}-1)\;{\left(\sqrt{1-x}\;\right)}^{n}\right),\;x\in [0,1),$$

and extend *F*_*n*_ to the real line using the periodicity condition *F*_*n*_(*x* + *m*) = *F*_*n*_(*x*) + *m*, m∈Z. Taking *c*_1_ = (*nb* − 2*c*)/((*n* − 1)*b*) gives *F*_*n*_(0) = *a*, $F_{n}^{\prime }(0)=c$ and lim _*x*↑1_*F*_*n*_(*x*) = *a* + *b*. The assumption *b* ∈ (0, 1) gives a discontinuity at x=m∈Z. Since

4.2 $$F_{n}^{\prime }(x)=\frac{b}{2\sqrt{1-x}}\;\left(c_{1}+(1-c_{1})n{\left(\sqrt{1-x}\;\right)}^{n-1}\right),$$

*F*_*n*_ is the lift of a monotonic circle map if 0 < *c*_1_ ≤ *n*/(*n* − 1), i.e. 0 ≤ *c* < *nb*/2. Note that at *c* = *nb*/2, *c*_1_ = 0 and the square-root singularity disappears. The lift *F*_*n*_(*x*) is increasing in the parameter *a*. A detailed analysis of the map is in the electronic supplementary material.

**Figure 6.**
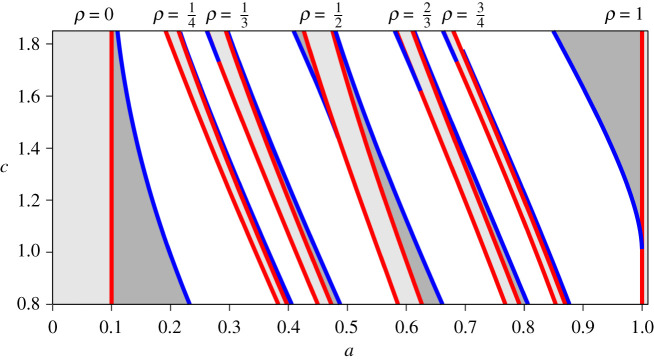
The bifurcation set in the *a*–*c* plane for the map *F*_*n*_(*x*) up to the fourth iterate. Parameters: *b* = 0.9, *n* = 5, *c* ∈ [0.8, 1.85]. The light/dark shaded regions are regions where one/two fixed points exist with the labelled rotation number. Transitions between different numbers of fixed points are either saddle-node bifurcation curves (blue) or border collisions (red). For each pair of border collision curves, the right curve is the type I border collision and the left curve is the type II border collision. (Online version in colour.)

In figure 6, the saddle-node bifurcation and border collision curves associated with the map and its first four iterates in the *a*–*c* plane for *b* = 0.9, *n* = 5 are depicted. It illustrates the two possible types of fundamental bifurcation sequences and the smooth change from the bifurcation sequence (a) to the bifurcation sequence (b). The transition occurs when (*F*^*q*^)′(0^+^) = 1 and the saddle-node curve merges with the type II border collision curve. Furthermore, it shows

— Every phase-locked region is always bounded by a saddle-node curve on the right.
— The border collision on the right is associated with a type I border collision with an infinite derivative and hence cannot merge with the right saddle-node curve.
— The border collision on the left is associated with a type II border collision with a finite derivative. For the smaller values of *c* (the derivative of *F*_*n*_′ at 0^+^), this is also the left bound of the phase-locked region.
— For some phase-locked regions, the left bound for the larger *c* values is formed by a saddle-node curve. This curve merges with the type II border collision curve when *c* decreases.
— The function *F*_*n*_ is convex for *c* ∈ [0, *bn*/2(*n* − 1)] = [0, 0.5625] and only the bifurcation sequence (a) occurs for those parameter values.

### (b) Example: the sinusoidal threshold system

Next, we continue with our illustrative example of a threshold system, the STS given by equations (2.2)–(2.3). As discussed in §3, this system has an associated circle map with a gap if the flow is tangent to the upper threshold on contact. This will happen if *h*′(*x*) > *γ*, i.e. *α* > *γ*. Thus, increasing *α* smoothly changes the map from continuous to a map with a gap. This enables us to see how the familiar saddle-node bifurcation structure for smooth maps without gaps, as shown in figure 2, transitions to the bifurcation sequences: first (b) saddle-node bifurcation → border collision → border collision → saddle-node bifurcation and then (a) border collision → border collision → saddle-node bifurcation.

Recall that the sufficient conditions for a saddle-node bifurcation curve of a (*p*, 1) fixed point are $\beta =p\tilde{\gamma }$ or $\beta =p\tilde{\gamma }-\alpha /\pi$. We will see now why this condition is only sufficient. An explicit expression for the infinite derivative type I border collision of a (*p*, 1) fixed point can be derived (recall that $\tilde{\gamma }=\gamma /(\gamma +1)$):

$$\alpha =\frac{\gamma^{2}+4\pi^{2}{\left(\tilde{\gamma }p-\beta \right)}^{2}}{4\pi (\tilde{\gamma }p-\beta )},\;\text{for}\;\max\left(0,p\tilde{\gamma }-\frac{\gamma }{2\pi }\right)\leq \beta <\tilde{\gamma }p.$$

This curve is a monotonically increasing function of *β* starting at the minimum value of $\beta =\tilde{\gamma }p-(\gamma /2\pi )$, *α* = *γ*, where *x*_*n*_ = *x*_*n*+1_ = 1 and asymptoting to the saddle-node line $\beta =\tilde{\gamma }p$ where *x*_*n*_ = *x*_*n*+1_ = 3/4.

The finite derivative type II border collisions for (*p*, 1) fixed points satisfy

$$\begin{array}{rl} & \cos2\pi x_{b}=\frac{\gamma }{\alpha },\\ & \sin2\pi x_{c}=\frac{2\pi }{\alpha }(p\tilde{\gamma }-\beta )-1\\ \text{and}\; & \sin2\pi x_{c}-\sin2\pi x_{b}=\frac{2\pi \gamma }{\alpha }(x_{c}-x_{b}), \end{array}$$

where *x*_*c*_ and *x*_*b*_ are equivalent to the time points on the periodic cycle indicated by *b* and *c* in figure 3. Note that *x*_*b*_ = *x*_*c*_ when *α* = *γ* and $\beta =p\tilde{\gamma }-\gamma /2\pi$, at which point the type I and type II border collisions coalesce. To find the intersection of the type II border collision curve and the saddle-node curve, we observe that *x*_*c*_ = 1/4 along the saddle-node curve $\beta =p\tilde{\gamma }-(\alpha /\pi )$. Substituting this into the equations gives the relation $1+\sqrt{1-q^{2}}=q((\pi /2)+\arccos(q))$, where *q* = *γ*/*α*. This has a unique solution q* ∈ (0, 1), q* ≈ 0.725. Hence, when *α* = *γ*/q* and $\beta =p\tilde{\gamma }-\gamma /q^{*}\pi$, then the type II border collision curve and the saddle-node curve collide and the border collision curve terminates the saddle-node curve. This implies that at *α* = *γ*/q*, the bifurcation sequence (b) transitions to the sequence (a).

**Figure 7.**
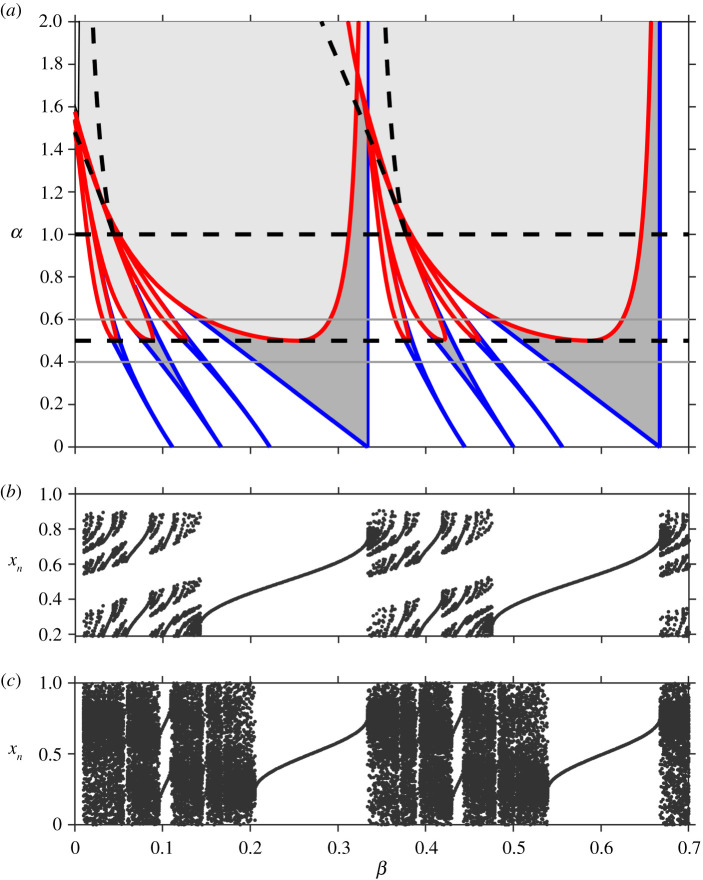
(*a*) Bifurcation set for *γ* = 0.5 showing the relation between border collisions (red) and saddle-node bifurcations (blue). Border collisions to the left-hand side of each minimum are of type II and to the right-hand side are of type I. The dashed horizontal line at *α* = *γ* = 0.5 marks the transition from continuous to gap map. The dashed horizontal line at *α* = 1 marks the transition from monotonicity to non-monotonicity. The dashed lines forming the ‘v’ shape mark the transition from single to multiple gaps, see §5. For *α* < 1, the light/dark shaded regions correspond to regions of existence of one stable/a pair of fixed points. For *α* > 1, the map is non-monotonic and the dynamics can be more complicated. In this region, period-doubling bifurcations also exist (not shown). (*b*) Bifurcation diagram showing stable solutions for *γ* = 0.5, *α* = 0.6 (corresponding to the upper light grey line in (*a*)). The gaps in the map appear as bands of ‘forbidden’ regions in the bifurcation diagram and result in the Cantor structure for quasi-periodic solutions. (*c*) Bifurcation diagram showing stable solutions for *γ* = 0.5, *α* = 0.4 (corresponding to the lower light grey line in (*a*)). The numerical bifurcation diagram has dark bands corresponding to the fact that there exist quasi-periodic solutions that densely fill the circle. (Online version in colour.)

Border collisions for general (*p*, *q*) solutions can be found numerically by solving the fixed point condition (2.6) along with the requirement that the fixed point occurs at the appropriate endpoint of the gap. In figure 7*a*, the bifurcation set for *γ* = 0.5 is shown for the first few iterates of the map. Between the lines *α* = *γ* = 0.5 and *α* = 1, the circle map is monotonic with a gap. In this region, the border collisions form u-shaped regions inside each tongue. The left-/right-hand side of each u-shaped region corresponds to the type II/type I border collision bifurcations, respectively. This illustrates how the sequences of bifurcations seen in continuous circle maps transition to the sequences seen in maps with gaps. The vertical derivative present in the threshold maps implies that one side of the saddle-node tongues is terminated by a border collision and the other side persists.

For *α* > 1, the map is non-monotonic. We consider the general transition from monotonic to non-monotonic maps in §5 and then continue with this example.

## 5. Tangencies leading to non-monotonic maps

**Figure 8.**
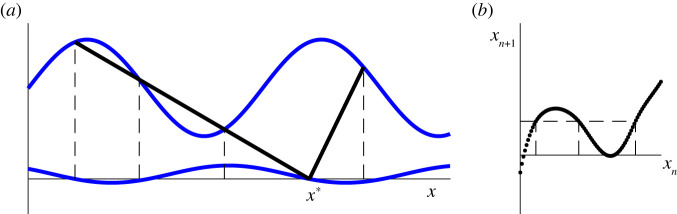
(*a*) The point *x* = 0 has multiple pre-images in the down map *T*_*d*_, leading to non-monotonicity in the associated threshold system circle map, see (*b*). (Online version in colour.)

**Figure 9.**
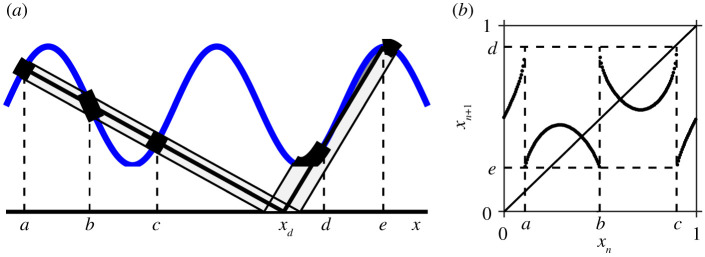
A tangency point between the downflow and the upper threshold leads to multiple pre-images, as shown in (*a*) for the STS (*α* = 4, *β* = 0.5, *γ* = 3). If there is also a tangency between the upflow and the upper threshold, then the corresponding threshold map has multiple gaps, where each gap has the same size with an infinite derivative on one side (at *x*_*n*+1_ = *d*) and a finite derivative on the other (at *x*_*n*+1_ = *e*). (Online version in colour.)

We return to the general threshold maps. We have seen in §3 that tangencies of the upflow with the upper threshold create discontinuities in the map. In this section, we will show that tangencies of the downflow with the upper threshold lead to multiple pre-images (non-monotonicity) in the down map *T*_*d*_ (see figure 8). This implies that tangencies of the upflow with the lower threshold lead to non-monotonicity in the up map *T*_*u*_. We will also discuss how and when tangencies in the upflow or downflow imply non-monotonicity in the full circle map. Finally, we will discuss how simultaneous tangencies in the up and down maps lead to the combination of gaps and non-monotonicity. This can lead to the presence of multiple gaps (see figure 9) and give rise to a codimension 2 bifurcation which organizes the local bifurcation structure. We illustrate the mechanism and the consequent bifurcations with the STS example.

### (a) Existence of non-monotonicity

As in §3, we consider a family of parametrized threshold maps with P the parameter space. The threshold maps are the composition of two maps: the down map $T_{d}:R\times P\to R$ from the upper boundary to the lower boundary and the up map $T_{u}:R\times P\to R$ from the lower boundary to the upper boundary. A map is monotonic if every point in its range has exactly one pre-image. By using the backward flow, we can find the pre-images of the down map. Let us consider the function

5.1 $$\tilde{W}(\tau ,x,\mu )=\psi_{-\tau }(g(x,\mu ),\mu )-h(x-\tau ),$$

i.e. $\tilde{W}$ is very similar to *W* as defined in (3.1), but uses the backward downflow starting at the lower threshold *g*(*x*, *μ*). If (τ*, x*, μ*) satisfies $\tilde{W}(\tau^{*},x^{*},\mu^{*})=0$, then x* − τ* is a pre-image of x* for the down map *T*_*d*_. Using the convention for derivatives from §3, if also ${\tilde{W}}_{1}(\tau^{*},x^{*},\mu^{*})=0$, then there is a tangency between the downflow $\psi_{\tau }(h(x,\mu ),\mu )$ and the upper threshold *h*(*x*, *μ*) at *x* = x* − τ*, *μ* = μ* and *τ* = 0. Due to the similarity between $\tilde{W}$ and *W*, the results of §3 give the local behaviour near a pre-image.

**Proposition 5.1.**

*Let* (τ*, x*, μ*) *satisfy* $\tilde{W}(\tau^{*},x^{*},\mu^{*})=0$, hence x* − τ* is a pre-image for x* *under T*_*d*_. *Assume the non-degeneracy conditions* ${\tilde{W}}_{2}(\tau^{*},x^{*},\mu^{*})\neq 0$ *and* ${\tilde{W}}_{3}(\tau^{*},x^{*},\mu^{*})\neq 0$, *then we have the following results*.

— *If* ${\tilde{W}}_{1}(\tau^{*},x^{*},\mu^{*})\neq 0$, *then for* (*x*, *μ*) *nearby* (x*, μ*), *T*_*d*_ *has a locally unique pre-image*.
— *If* ${\tilde{W}}_{1}(\tau^{*},x^{*},\mu^{*})=0$ *and* ${\tilde{W}}_{11}(\tau^{*},x^{*},\mu^{*})\neq 0$, *then there is a fold along a curve in the* (*x*, *μ*)-*plane given by* ${\tilde{W}}_{2}x+{\tilde{W}}_{3}\mu =0$ *in lowest order. The fold has again two interpretations: it represents a unique pre-image for the map T*_*d*_ *along the fold line. And in the threshold system, it gives also a persisting simple tangency between the upwards trajectory and the lower threshold at this point. When* ${\tilde{W}}_{11}({\tilde{W}}_{2}x+{\tilde{W}}_{3}\mu )<0$, then there are two pre-images, one less and one greater than x* − τ*. *Both pre-images are relevant for the map T*_*d*_, *which has a turning point at the unique pre-images on the fold line, i.e. at the tangency points; see figure 8*.
— *If* ${\tilde{W}}_{1}(\tau^{*},x^{*},\mu^{*})=0={\tilde{W}}_{11}(\tau^{*},x^{*},\mu^{*})$ *and* ${\tilde{W}}_{111}(\tau^{*},x^{*},\mu^{*})\neq 0$, *then again generically we have a cusp unfolding and locally there is a change in monotonicity with two turning points emerging in the map T*_*d*_.

As *T*_*d*_ is periodic, this local analysis shows that globally the map *T*_*d*_ always has an even number of tangency points and an odd number of pre-images (counting multiplicity at the degenerate points).

### (b) Simultaneous tangencies

We have now shown for the up and down maps that tangencies between the flow and the nearby threshold correspond to non-monotonicity and tangencies between the flow and the opposite threshold correspond to discontinuities in those maps (§3). The threshold map is a composition of the up and down maps; hence, these tangencies will influence the monotonicity and continuity of the threshold map. The derivative of the threshold map is

$${\left(T_{u}\circ T_{d}\right)}^{\prime }(x)=T_{u}^{\prime }(T_{d}(x))\;T_{d}^{\prime }(x).$$

Thus, a tangency between the downflow and the upper threshold leads to a turning point in the threshold map. And a tangency between the upflow and the lower threshold leads to a turning point in the threshold map if this tangency occurs at a point that is in the range of the down map.

If both the up and down map have tangencies with the upper threshold, then the result will be a non-monotonic, discontinuous map. The presence of both gaps and non-monotonicity can also lead to further structural changes in the map: a transition from a single gap to three (or more) gaps. The transition to multiple gaps occurs as follows (see also figure 9). Suppose that the upflow is tangent to the upper threshold at a first intersection point at *x* = *d*. This leads to a gap in the up map *T*_*u*_, say at *x* = *x*_*d*_, i.e. $T_{d}(x_{d}^{-})=d$ and $T_{d}(x_{d}^{+})=e$ for some *e* > *d*. When the down map is monotonic (i.e. has no tangencies between the downflow and the upper threshold), the point *x* = *x*_*d*_ will have a single pre-image in the down map and there will be a single gap in the threshold map. When the down map is non-monotonic, the point *x* = *x*_*d*_ can have three (or more) pre-images, as illustrated in figure 9.

In the corresponding circle map, there will be a gap associated with each of these pre-images. In each case, the gap arises from the same tangency; thus, the size and the qualitative nature of the gap will be preserved. The unbounded derivative can occur either to the left or the right of the gap depending on the slope of the upper threshold at the pre-image.

The transition point between one and three gaps occurs when the down map has a tangency with the upper threshold and this tangency point is mapped into *x* = *x*_*d*_.

**Figure 10.**
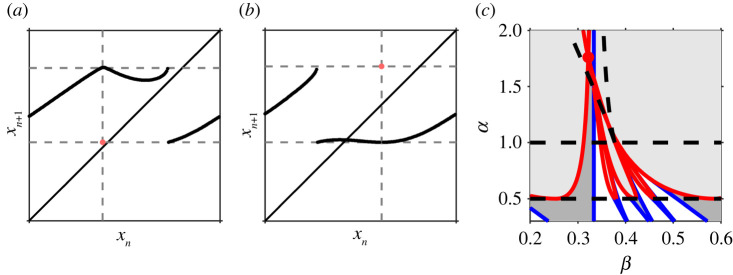
(*a*) Map on the left-hand edge of the v-shaped wedge for *α* = 1.3, *β* = 0.3508, *γ* = 0.5. This left-hand edge corresponds to the point when the local maximum coincides with the side of the gap with infinite derivative. The orange dot denotes the isolated point in the map. (*b*) Map on the right-hand edge of the v-shaped wedge for *α* = 1.3, *β* = 0.3653, *γ* = 0.5. (*c*) Bifurcation set for the STS for *γ* = 0.5 showing a blow-up of the v-shaped region. (Online version in colour.)

In the notation of figure 9, at this special point, we have *b* = *c* and the threshold map maps *b* into *d*. This is an isolated point in the map as all points nearby *b* get mapped nearby *e*. The finite derivative at *e* vanishes and there is no derivative at *d* as it is an isolated point. How the creation of multiple gaps plays out in the STS circle map is illustrated in figure 10. The two points at which tangencies with the upper threshold map into *x* = *x*_*d*_ correspond to a local maximum and a local minimum of the circle map. These tangencies are mapped into *x* = *x*_*d*_ when the local maximum coincides with the infinite derivative (see figure 10*a*) or the local minimum coincides with the finite derivative (see figure 10*b*). The isolated point in both cases is marked in orange.

Tangencies in both the upflow and downflow with the lower threshold do not lead to multiple gaps. This apparent asymmetry is a consequence of the fact that we have considered the map from the upper threshold to the upper threshold ($T_{u}\circ T_{d}$). Generically, the tangency of the down map with the lower threshold creates a gap; this gap persists under the action of the up map and hence creates a gap in the threshold map. Non-monotonicity of the up map (tangencies of the upflow with the lower threshold) does not affect the pre-images of the gap. In this case, the only mechanism in which multiple gaps can be created is by non-monotonicity of the down map, i.e. the downflow being tangent simultaneously to the upper and lower threshold. Thus, the multiple gaps in the threshold map reflect the multiple tangencies in the down map. In §5d, we discuss further the influence of the order of composition on the apparent structure of the map.

The consequences of non-monotonicity for bifurcations in the standard circle map are discussed in [19]. All the typical features of period-doubling bifurcations and the associated transition to chaos can be seen in threshold systems too.

### (c) Example: the sinusoidal threshold system

We continue with the STS example given by equations (2.2)–(2.3). As the lower threshold is flat, there is no tangency between the upflow and the lower threshold, hence the up map *T*_*d*_ is monotonic and the down map *T*_*d*_ is continuous. Thus, the STS map is monotonic if and only if the down map *T*_*d*_ is monotonic, i.e. if and only if *h*′(*x*) ≤ 1 for all *x*, which is equivalent to 0 < *α* ≤ 1. Transitions from one to three gaps occur when both the upflow and downflow have tangencies with the upper threshold and the tangency point of the downflow with the upper threshold is mapped into the *T*_*u*_ pre-image of the tangency point of the upflow with the upper threshold.

These transitions can be computed numerically and are shown for the particular case *γ* = 0.5 by the v-shaped curves formed by the dashed lines in figures 7*a* and 10*c*. Even though there are three gaps, no new border collision curves will be formed. This follows from the observation that the fixed points and bifurcation curves of the maps $T_{u}\circ T_{d}$ (mapping upper threshold into upper threshold) and $T_{d}\circ T_{u}$ (mapping lower threshold into lower threshold) are equivalent. The latter map is the composition of a non-monotonic, continuous map acting on a monotonic, discontinuous map. So, the gap has a unique pre-image, implying that there are at most two border collision curves, see also the section below.

**Figure 11.**
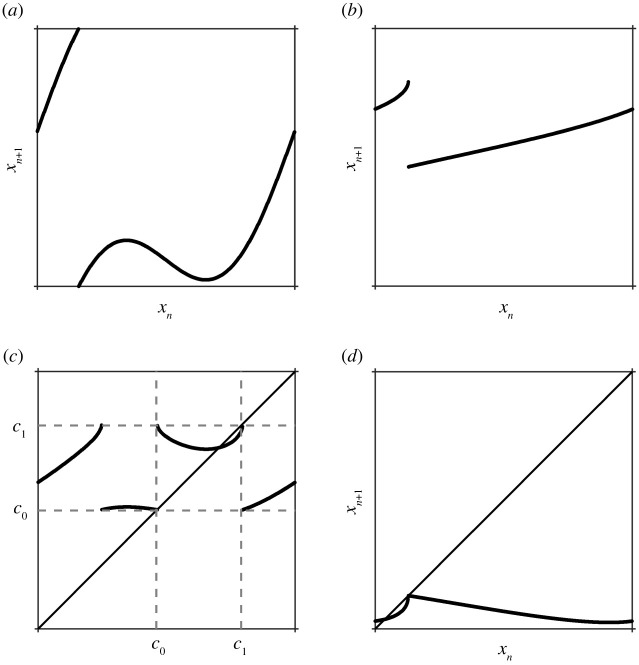
Maps at the intersection point of the border collisions, the point which is marked by a red dot in figure 10*c*. (*a*) The down map *T*_*d*_ which is non-monotonic because of a tangency of the downflow with the upper threshold. (*b*) The up map *T*_*u*_ which contains a gap as a consequence of a tangency of the upflow with the upper threshold. (*c*) $T_{d}\circ T_{u}$. (*d*) $T_{u}\circ T_{d}$.

Figure 10*c* shows that the type I border collisions for the (1, 1)-tongue and the type II border collisions for the (2, 1)-tongue cross in the three-gap region (inside the v-shaped region). The map at this point is shown in figure 11*c*. The two border collision points are denoted by *c*_0_ (type II border collision) and *c*_1_ (type I border collision), i.e. the tangency between the upflow and the upper threshold is at *c*_1_. This implies that there is some *x*_*d*_ such that $T_{u}(x_{d}^{-})=c_{1}$, $T_{u}(x_{d}^{+})=c_{0}$ and *T*_*d*_(*c*_0_) = *x*_*d*_ = *T*_*d*_(*c*_1_). Many of the border collisions from the intermediate tongues appear to converge on this crossing point, suggesting that it forms a codimension two point that organizes the local bifurcation structure. In the next section, we will show that this is indeed the case.

Though we will not go into the details here, we note that explicit expressions can be derived for the first period-doubling bifurcation for (*p*, 1) fixed points, giving rise to fixed points (2*p*, 2). The case *γ* = 3 is particularly interesting, since at this value, the period-doubling and type I border collision curves coincide.

### (d) A codimension two bifurcation

In this section, we conjecture that if the upflow has a generic tangency with the upper threshold and the down map maps both end points of this gap into the pre-image of the up map associated with the tangency, then the corresponding codimension 2 border collision bifurcation point is an organizing centre for the local bifurcations. Introducing the parameter vector $\mu \in R^{2}$, such codimension 2 point is characterized by the following properties. There exist *c*_0_, *c*_1_, *c*_0_ ≠ *c*_1_ such that

$$\begin{array}{rl} & T_{u}(x_{0}^{-};\text{0})=c_{0};\;T_{u}(x_{0}^{+};\text{0})=c_{1};\\ & T_{d}(c_{1};\text{0})=T_{d}(c_{0};\text{0})=:x_{0};\;T_{d}^{\prime }(c_{1};\text{0})\;T_{d}^{\prime }(c_{0};\text{0})<0. \end{array}$$

This implies that the threshold map $T_{u}\circ T_{d}$ has multiple gaps, and there are simultaneously two border collisions: one type I border collision at *c*_0_ and one type II border collision at *c*_1_. To analyse this border collision, it is convenient to consider the map from the lower threshold to the lower threshold, i.e. $T_{d}\circ T_{u}$. This map has the same number of fixed points and bifurcations as the threshold map $T_{u}\circ T_{d}$. Under the conditions above, the border collisions in the map $T_{d}\circ T_{u}$ collide when μ=0. The map is continuous at *x*_0_ and the derivatives on each side have opposite signs (with one of them having a square-root singularity).

In §3, it is shown that the gap in *T*_*u*_ persists for μ small and that generically the derivative of *T*_*u*_ nearby the gap has the same sign at both sides of the gap (with one of them having a square-root singularity). For μ≠0, the discontinuity in *T*_*u*_ leads to a discontinuity in the threshold map $T_{d}\circ T_{u}$ with the derivatives at each side of the gap still having opposite signs. Hence, the parameter plane nearby μ=0 can be divided into four regions which are such that the threshold map has two solutions in one region, one solution in two regions and no solutions in one region. In [24, §7.1.1], it is shown that if the local derivatives are less than 1 (i.e. the map is contracting) such maps are organizing centres for the local bifurcations. It is also noted that this bifurcation point is equivalent to the *gluing bifurcation* in [31,32] and *big bang bifurcation* in [26]. Although we do not satisfy the condition that the derivatives are less than 1 (there is a square-root singularity at one of the end points), we still see the organizing centre in the bifurcation diagram. Avrutin *et al*. [33] have studied maps on the real line with similar singularities in the derivative, although our local behaviour does not seem to be one of the cases they study in detail. Our behaviour looks more like a one-dimensional Nordmark map at the grazing point.

## 6. Other mechanisms: Cherry flows

The creation of gaps in circle maps arises naturally in other contexts. If two oscillators interact then a lowest order model might relate the evolution of the phase of each oscillator. In this case, the natural phase space is the torus (one angle for each phase) leading to a differential equation on the torus. Examples include neuronal models such as the Kuramoto equations [34] and models of breathing patterns [10].

Suppose that a flow on the torus has a homotopically nontrivial transverse section. Then, the return map on the global section is a circle map as discussed in previous sections. There are two natural classes [9]. In a Poincaré flow, this map is continuous and monotonic, so the classic results about the existence of rotation numbers and the dichotomy of dynamics depending on whether the rotation number is rational or irrational hold. On the other hand, a Cherry flow [9,12,35] has at least one unstable stationary point and one saddle, which can create a return map which is monotonic and with a discontinuity. Such maps have a well-defined rotation number, and for continuous perturbations of the defining vector field, this rotation number varies continuously (see §1 and [22,23]). In particular, if the family of maps has parameter values which have rotation numbers that are different, then there are parameters with irrational rotation numbers. Since the image of the cross-section is not surjective because of the discontinuity this is a natural way to construct a Denjoy counterexample (a map with an irrational rotation number but no dense orbits).

Cherry flows are classic examples from geometric dynamics [12], and the transition from a Poincaré flow to a Cherry flow has been discussed briefly in [27]. In this section, we give a brief account of the scalings predicted by a theoretical model of this transition and describe a piecewise-smooth example. We will show that unlike for the threshold systems considered in §3, the size of the discontinuity does not tend to zero at the transition point, and that at the discontinuity the slope of the map tends to infinity with a characteristic scaling. The first of these results is noted in [27, p. 423].

A pair of stationary points can be created in a saddle-node bifurcation. Suppose that $\tilde{\mu }$ is a real parameter and that if $\tilde{\mu }>0$ there are no stationary points of the flow, while if $\tilde{\mu }<0$ there is an unstable stationary point and a saddle. Then, there are local coordinates (*ξ*, *η*) such that in a neighbourhood of $\left(\xi ,\eta ,\tilde{\mu })=(0,0,0\right)$ the leading order terms of the differential equation are

6.1 $$\begin{array}{rl} & \dot{\xi }=\mu +\xi^{2}\\ \text{and}\; & \dot{\eta }=\lambda \eta \end{array}\}$$

with *λ* > 0 and *μ* is a rescaled version of the original parameter $\tilde{\mu }$. We would like to derive a leading order return map through a neighbourhood of the origin from *ξ* < 0 to *ξ* > 0 which can then be composed with the standard return maps for Poincaré type flows away from this singularity to obtain a theoretical model of the global return map for the transition from a Poincaré flow to a Cherry flow.

In the classic form (6.1), the unstable manifolds of the stationary points are vertical, and so it is not possible to define a return map from positive to negative *ξ*. This suggests that for this problem we should add a further change of coordinates

6.2 $$x=\xi +a\eta^{2},\;y=\eta ,\;(a>0),$$

so that in these new coordinates, the unstable manifold of the saddle-node stationary point at *μ* = 0 is *x* = *ay*^2^, *ξ* = 0 in (6.2), making a return map from negative *x* to positive *x* possible even if *μ* < 0. Another way of seeing this is to claim that generically the unstable manifolds are parabolic curves to the lowest order at the saddle-node bifurcation, and this modification of coordinates has the effect of making the unstable manifolds quadratic functions without complicating the underlying dynamics.

In the new coordinates (6.1) becomes

6.3 $$\begin{array}{rl} & \dot{x}=\mu +x^{2}+2a\lambda y^{2}-2axy^{2}+a^{2}y^{4}\\ \text{and}\; & \dot{y}=\lambda y. \end{array}\}$$

As with (6.1), (6.3) is the leading order approximation of the vector field in a neighbourhood N of the origin in phase space and parameter space, i.e. |*x*|^2^ + |*y*|^2^ + |*μ*|^2^ < ε^2^ for some small ε > 0.

Fix ε > 0 and N as above and let *k* be a constant, 0 < *k* < 1 to be determined. Our goal is to derive a return map of (6.3) from *x* = −*k*ε to *x* = *k*ε in N. This can then be embedded in a smooth flow that defines a return map which is locally just a smooth deformation of this map. If the initial condition is ( − *k*ε, *y*_0_) in N then this corresponds to $\left(\xi_{0},\eta_{0})=(-k\varepsilon -ay_{0}^{2},y_{0}\right)$ and since *a* > 0, *ξ*_0_ < 0. Suppose that *μ* > 0, so we can write

$$\mu =\sigma^{2},\;\sigma >0.$$

Solutions to (6.1) are *ξ* = *σ*tan (*σt* + *C*), *η* = *η*_0_exp (*λt*) or

$$x=\sigma \tan(\sigma t+C)+ay_{0}^{2}\exp(2\lambda t),\;y=y_{0}\exp(\lambda t).$$

The initial condition ( − *k*ε, *y*_0_) implies that $\sigma \tan C+ay_{0}^{2}=-k\varepsilon$ and so as *σ* → 0

$$C=-\frac{\pi }{2}+\frac{\sigma }{k\varepsilon +ay_{0}^{2}}+O(\sigma^{2}).$$

Provided it stays in N, this solution intersects *x* = *k*ε after time *T* given by

6.4 $$\sigma \tan(\sigma T+C)+ay_{0}^{2}\exp(2\lambda T)=k\varepsilon .$$

If |*y*_0_| is sufficiently small so that the first term on the left-hand side of (6.4) dominates the second term this leaves *σ*tan (*σT* + *C*) ≈ *k*ε, so

$$T\approx \frac{\pi }{\sigma }+O(1),$$

and *T* → ∞ as *σ* ↓ 0.

This approximation holds in an exponentially small region of parameter space with |*y*_0_| ≪ εexp ( − *λT*). However, in this very small neighbourhood of *y*_0_ = 0, the return map through the region where the saddle-node bifurcation is about to take place is approximately

$$y\to \exp(\lambda T)y\approx {\left({\text{e}}^{\lambda \pi }\right)}^{1/\sigma }y.$$

In other words, there is a small neighbourhood on which the slope *s* of the map is very steep and for *μ* → 0^+^ tends to infinity with

$$\log\;s\approx \frac{\lambda \pi }{\sigma }=\frac{\lambda \pi }{\sqrt{\mu }}.$$

Note that the constant *λπ* is determined by the normal form (6.1), and in general, $\log\;s\approx \kappa /\sqrt{\mu }$ for some constant *κ*.

Now suppose that *μ* < 0, so

$$\mu =-\sigma^{2},\;\sigma \geq 0.$$

The derivation of the return map is more standard in this case. By construction, there are stationary points at ( ± *σ*, 0) and ( − *σ*, 0) is a saddle. The unstable manifold of the saddle in (*x*, *y*) coordinates is the curve

$$x=-\sigma +ay^{2}$$

and so this intersects *x* = *k*ε at $y=\pm \sqrt{(k\varepsilon +\sigma )/a}$. This implies that if *σ* ≥ 0, there is an interval of length $2\sqrt{(k\varepsilon +\sigma )/a}$ between the two intersection points of the unstable manifold with *x* = *k*ε which cannot be reached by trajectories starting on *x* = −*k*ε in N. At the bifurcation value *σ* = 0, this is non-zero and so the limit of the size of the discontinuity of the return map does not tend to zero at the bifurcation value [27]. Moreover, standard analysis (e.g. [36]) close to the saddle shows that the slope of the return map at the discontinuity tends to infinity as the leading order non-constant term of the return map is

$$C_{1}|y{|}^{\alpha },\;\alpha =\frac{2\sqrt{|\mu |}}{\lambda }<1.$$

Very briefly, the argument is that the saddle which exists if *μ* < 0 has linearized eigenvalues *λ* > 0 and 2x*, where $x^{*}$ is the *x*-coordinate of the saddle point, i.e. $x^{*}=-\sqrt{|\mu |}$. Working in new coordinates (*x*, *y*), with the obvious abuse of notation, centred on (x*, 0) the flow is locally approximately linear and so solutions are locally $x=x^{*}+c\;{\text{e}}^{-2\sqrt{|\mu |t}}$, *y* = *y*_0_*e*^*λt*^. A solution starting at (x* − *h*, *y*_0_) strikes *y* = ±*h* after a time $t=\frac{1}{\lambda }\log(h/y_{0})$ and the corresponding *x*-coordinate is $x^{*}-h\;{\text{e}}^{-2\sqrt{|\mu |t}}\approx x^{*}-Ky_{0}^{2\sqrt{|\mu |}/\lambda }$. Thus, distances from the invariant manifolds change from *y* (close to the stable manifold) to *y*^*α*^ (close to the unstable manifold) as solutions pass through a neighbourhood of the saddle.

To summarize the theoretical predictions, we have

— if *μ* > 0 then as *μ* ↓ 0 the return map develops an exponentially small region on which the slope *s* of the return map grows large and scales with $\log s\approx \frac{\kappa }{\sqrt{\mu }}$ for some constant *κ*;
— if *μ* ≤ 0 then the return map has is a finite discontinuity and if *μ* < 0 then the slope of the map tends to infinity at the discontinuity.

**Figure 12.**
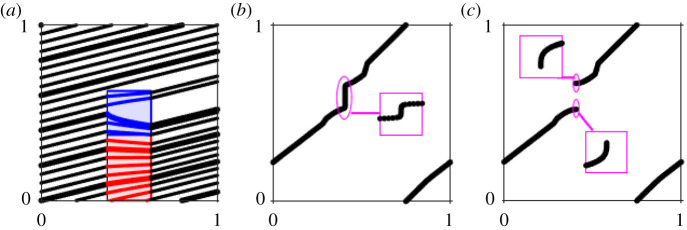
The piecewise-smooth model of the transition to a Cherry flow with parameters (6.5). (*a*) Flow with *μ* = 1/70 showing a stable solution that winds many times around the torus. Here, the blue square is a translated version of system (6.3). Together with the flows defined in the red and black regions, this gives a flow on the torus $T^{2}={\left[0,1\right]}^{2}$. The full equations for each region are given in the electronic supplementary material. (*b*) Return map on *x* = 0 for *μ* = 1/70 with inset showing the region with high derivative (*x*_*n*_ ∈ [0.406245, 0.406255], *x*_*n*+1_ ∈ [0.45, 0.7])); and (*c*) return map on *x* = 0 for *μ* = −1/70 with insets showing the regions around each end of the discontinuity (*x*_*n*_ ∈ [0.4061, 0.4064] with *x*_*n*+1_ ∈ [0.6674, 0.6677] for the upper end and *x*_*n*+1_ ∈ [0.5198, 0.5201]) for the lower end). (Online version in colour.)

The system (6.3) could be smoothly embedded in a global flow to create a *C*^∞^ example of the transition from a Poincaré flow to a Cherry flow that could be analysed numerically. To simplify the construction and to make it possible to calculate some quantities explicitly, we have chosen to use a piecewise linear global flow around the nonlinear saddle-node bifurcation. The details of this embedding can be found in the electronic supplementary material. Figure 12 illustrates the flow and return maps associated with this model as *μ* passes through zero. The parameters used are as follows:

6.5 $$a=45,\;\mu =\pm \frac{1}{70},\;k\varepsilon =\frac{1}{8}.$$

With these parameters, a gap of size $2(1/\sqrt{8\times 45})\approx 0.1054$ opens up between the intersections of the local unstable manifolds of the non-hyperbolic stationary point on the line *x* = 5/8 as *μ* decreases through zero and increases in size as *μ* decreases.

Figure 12*a*,*b* shows the flow and the associated return map with *μ* = 1/70, i.e. when the flow is still a Poincaré flow. The return map clearly displays the very steep derivative over a significant region in the *x*_*n*+1_ variable, but a very small region in the *x*_*n*_ variable. The inset shows a blow-up of the map in a segment of *x*_*n*_ values containing the region of high derivative. This emphasizes just how narrow the regions involved become and illustrates the rapid transition between shallow and steep derivatives.

Figure 12*c* shows the associated return map with *μ* = −1/70, at which the flow is a Cherry flow. For most *x*_*n*_ values, the return map is almost identical, except that the steep curve has been replaced by a gap. The insets show a neighbourhood of the points of discontinuity. This reveals the infinite slope at the point of discontinuity.

## 7. Conclusion

In this paper, our focus has been to understand how structural transitions occur in maps derived from fundamental models. We have considered transitions from continuity to discontinuity, monotonicity to non-monotonicity and the creation of multiple gaps, and have described how these transitions can alter the bifurcations and dynamics of circle maps. Understanding how these structural transitions occur and their consequences suggests some new phenomena and gives a wider context within which to interpret some of the existing literature.

For example, in the study of maps with gaps, much of the focus has been on gaps where the derivatives at either side of the gap are bounded. Applications of such maps included threshold maps with non-smooth thresholds, like the combs in [4] or the triangles and rectangles in [1]. However, these non-smooth thresholds were introduced as approximations of smooth thresholds to allow for explicit calculations, and there are other applications (e.g. impact oscillators) in which finite derivatives do not occur [33,37]. Generically, we have shown that both in threshold systems and in the creation of Cherry flows one expects the associated discontinuous circle map to have a singularity in the derivative to one (threshold models) or both (Cherry flows) sides of the gap. In the case of threshold systems, it is the contact between the up/downflow and the upper/lower thresholds that is important in determining the local behaviour: if the contact is at a tangency, then generically a gap and the square-root singularity result. At the first tangency in a family of such maps, the size of the gap increases continuously from zero for the threshold models, but for Cherry flow, there is a discontinuous jump to a finite gap at the transition point. Another interesting example of transitions in circle maps initiated by tangencies in the underlying system can be found in the context of phase-resetting maps (see [38]). It was shown that before the transition from degree-one to degree-zero the phase-resetting map loses monotonicity via a tangency.

We have shown that the natural consequence of the square-root singularity is that one expects to see sequences of border collisions (types I and II of §4) interspersed with saddle-node bifurcations. Using a specific example threshold model, we have illustrated how the Arnold tongue bifurcation set for continuous monotonic circle maps with periodic solutions created and destroyed by saddle-node bifurcations transitions to a bifurcation set where periodic solutions can in addition be created/destroyed by border collisions. This underlying structure underpins the bifurcation sets found numerically by Glass *et al.* [2,13] and the recent work on the two-process model for sleep–wake regulation [39].

The transition from no gaps to gaps in piecewise-smooth monotonic maps also has an important consequence for non-periodic solutions. With no gaps, non-periodic solutions are quasiperiodic, and typically, they are dense in the circle. With gaps, solutions tend to a Cantor set [22]. Once noted, this difference is readily observable in numerically computed bifurcation diagrams, as illustrated in figure 7*b*,*c*.

For threshold systems, we have identified that the transition to non-monotonicity is also the result of tangency, this time of the up/downflow with the lower/upper threshold. The presence of both gaps and non-monotonicity gives many different new possibilities. For example, we have shown that there is a natural transition from circle maps with a single gap to multiple gaps. This in turn leads to a novel codimension two point in which there is the coincidence of two border collisions. A provisional analysis of this codimension two point shows how it acts as a local organizing centre, out of which an infinite sequence of other border collisions emerge (cf. [24]); details will be published elsewhere.

Threshold systems, even the simple example that we have chosen to illustrate many of our results, have extremely rich dynamics which we have not sought to classify exhaustively. Instead, we have shown how continuous (or not), injective (or not) circle maps sit within an overall framework with transitions between map types governed by tangencies. We have also highlighted how some generic novel higher codimension bifurcations can then occur.

## Data accessibility

This article has no additional data.
